# Japan’s contributions to hip joint preservation and reconstruction: from osteonecrosis and developmental dysplasia to precision arthroplasty

**DOI:** 10.1007/s00264-026-06957-2

**Published:** 2026-07-14

**Authors:** Masaki Takao, Wataru Ando, Takashi Sakai, Hidetoshi Hamada, Keisuke Uemura, Tatsuhiko Kutsuna, Takashi Imagama, Nobuhiko Sugano

**Affiliations:** 1 Department of Orthopaedic Surgery, Ehime University Graduate School of Medicine, Toon, Japan; 2 Department of Orthopaedic Surgery, Kansai Rosai Hospital, Amagasaki, Japan; 3 Department of Orthopaedic Surgery, Osaka University Graduate School of Medicine, Suita, Japan; 4 Department of Orthopaedic Surgery, Yamaguchi University Graduate School of Medicine, Ube, Japan; 5 Department of Orthopaedic Medical Engineering, Osaka University Graduate School of Medicine, Suita, Japan; 6 The Arthroplasty Center, Kawanishi City Medical Center, Kawanishi, Japan; 7 Department of Business Engineering, The University of Osaka Graduate School of Engineering, Suita, Japan

**Keywords:** Osteonecrosis of the femoral head, Developmental dysplasia of the hip, Hip preservation, Total hip arthroplasty, Computer-assisted surgery

## Abstract

**Purpose:**

To review Japan’s major contributions to hip joint preservation and reconstruction, spanning joint-preservingosteotomy, biomaterials innovation, and computer-assisted precision arthroplasty.

**Methods:**

A narrative review was performed to summarize landmark clinical and scientific advances originating from Japan in the management of osteonecrosis of the femoral head (ONFH), developmental dysplasia of the hip (DDH), total hiparthroplasty (THA), bearing surface technology, and computer-assisted surgery.

**Results:**

Japanese research has advanced the understanding and treatment of ONFH through nationwide epidemiological studies, development of classification systems, clarification of steroid-associated pathophysiology, and long-term validation of joint-preserving procedures, particularly transtrochanteric rotational osteotomy. In DDH, Japanese surgeons refined reconstructive osteotomies and established surgical strategies for complex primary and high-dislocation THA. Early clinical adoption of highly cross-linked polyethylene and advances in ceramic bearing technology improved implant longevity by reducing wear and osteolysis. Japan also pioneered CT-based navigation for both acetabular and femoral component placement and introduced the functional pelvic plane concept, shifting implant alignment from fixed safe zones toward individualized functional positioning that incorporates stem anteversion and spinopelvic dynamics. These innovations have subsequently expanded to robotic-assisted surgery and advanced three-dimensional reconstruction.

**Conclusion:**

Japan has made substantial contributions to modern hip surgery, from biological joint preservation to precision arthroplasty. This integrated approach—emphasizing anatomical accuracy, biomechanical restoration, and long-term durability—continues to influence contemporary practice and provides a foundation for future advances in robotics, artificial intelligence, and personalized hip reconstruction.

## Introduction

Hip surgery in Japan has evolved through a distinctive integration of biological preservation, three-dimensional anatomical understanding, precision engineering, and rigorous long-term clinical evaluation. Rather than focusing solely on prosthetic replacement, Japanese orthopaedic surgeons have historically emphasized preservation of native joint structures, individualized reconstruction, and meticulous assessment of hip morphology and function.

This philosophy has generated influential contributions in several domains, including the management of osteonecrosis of the femoral head (ONFH), the reconstruction of developmental dysplasia of the hip (DDH), innovations in bearing surface technology aimed at improving implant longevity, and the development of CT-based computer-assisted surgery for precision total hip arthroplasty (THA) and osteotomy.

In ONFH, Japanese investigators advanced diagnostic criteria, classification, and staging systems, clarified disease mechanisms, and pioneered joint-preserving surgical strategies that have influenced hip preservation surgery worldwide. In the field of DDH, Japanese surgeons established acetabular dysplasia as a major precursor of secondary osteoarthritis and developed reconstructive osteotomies based on detailed three-dimensional anatomical understanding, thereby advancing joint-preserving reconstruction. In parallel, research on highly cross-linked polyethylene, ceramic bearing surfaces, and retrieval-based evaluation contributed to improving long-term implant durability. Moreover, Japanese centres have played leading roles in three-dimensional preoperative planning, navigation-assisted surgery, and functional spinopelvic assessment, promoting the evolution of precision hip reconstruction.

In this narrative review, we summarize major Japanese contributions to hip surgery with particular focus on ONFH, DDH, bearing surface innovations, and CT-based computer-assisted techniques, highlighting conceptual frameworks and technological developments that have shaped contemporary orthopaedic practice.

## ONFH and joint preservation

ONFH has been a central focus of hip research in Japan for nearly five decades. Although the annual incidence in Japan is relatively low (approximately 2,000–3,000 new cases), the disease poses substantial clinical burden because it frequently affects young and middle-aged adults, often bilaterally, thereby creating a long lifetime demand for joint-preserving strategies or durable reconstruction. In 1975, the Japanese Ministry of Health and Welfare established a nationwide research group for ONFH, and the disease was subsequently designated as a specified intractable disorder. This framework enabled sustained epidemiologic surveillance and coordinated multicenter investigation, resulting in one of the most comprehensive longitudinal ONFH datasets worldwide [1, 2].

A hospital-based sentinel monitoring system involving specialized centres has provided continuous nationwide data with high diagnostic accuracy [3, 4]. These studies consistently demonstrate a male predominance (male-to-female ratio approximately 1.6–1.7:1). Age distribution differs by sex, with peaks in the sixth decades in men and in the seventh decade in women. Population-adjusted analyses have shown a decreasing incidence among younger individuals and a relative increase in middle-aged and older populations. Alcohol-associated ONFH accounts for roughly half of cases in men, whereas steroid-associated disease accounts for approximately 70% of cases in women. The proportion of systemic lupus erythematosus among steroid- associated cases has declined over time [3, 4]. Geographic variation in incidence has been observed, and heavy smoking has been identified as a significant risk factor in men, suggesting a potential role for modifiable lifestyle factors in disease prevention [5].

A defining contribution from Japan has been the development of rigorous diagnostic criteria and prognostic classification systems. Radiographic staging and lesion-type classification were first established in the 1980 s through the national research group [6]. Large-scale natural history studies subsequently demonstrated that the size and, critically, the location of the necrotic lesion within the weight-bearing region of the femoral head are decisive determinants of collapse. Lesions involving the lateral weight-bearing portion were shown to carry a markedly higher risk of structural failure than medial lesions [6].

The introduction and systematic application of magnetic resonance imaging (MRI) further transformed the field. Japanese investigators clarified early MRI features of ONFH, including the characteristic band pattern that precedes radiographic change and the frequent presence of asymptomatic contralateral involvement [7]. Three-dimensional MRI-based quantification demonstrated that lesion size and spatial distribution within the weight-bearing dome predict collapse more reliably than two-dimensional or angular measurements alone [8]. Longitudinal image registration techniques enabled dynamic assessment of lesion progression and repair, revealing that small medial lesions may stabilize, whereas lateral lesions commonly progress to collapse [9]. These insights culminated in the 2001 revised diagnostic criteria and the Japanese Investigation Committee (JIC) classification, which categorizes lesions according to their location within the weight-bearing surface [10]. The JIC system has shown superior predictive value for femoral head collapse and subsequent need for THA compared with alternative schemes and is now widely used internationally as a practical guide for treatment decision-making [11].

Beyond imaging and classification, Japanese studies have contributed to understanding the biological mechanisms underlying collapse and repair. Histopathological [12] and molecular investigations have demonstrated angiogenic and reparative responses within necrotic lesions, including expression of hypoxia-inducible factor-1α, vascular endothelial growth factor, and activation of osteoclast-related pathways [13]. Synovial inflammatory mediators have also been implicated in disease activity [14]. These findings support the concept that structural failure results from the balance between reparative processes and mechanical stress, providing a scientific rationale for lesion-specific prognostication and joint-preserving strategies.

Clinical practice patterns have evolved in parallel with these insights. Nationwide analyses indicate that the proportion of patients undergoing THA for ONFH increased from approximately 50% in the early 2000 s to nearly 80% in the mid-2010s, whereas osteotomy and bipolar hemiarthroplasty have declined [15]. The median time from definitive diagnosis to surgery is approximately nine months, underscoring the rapid progression of advanced disease [16]. Importantly, studies of patients diagnosed solely by MRI in the pre-radiographic stage demonstrate that fewer than half subsequently develop radiographic progression, highlighting the risk of overtreatment if intervention is based solely on early imaging findings [17].

Large multicenter arthroplasty registry studies have further identified risk factors for dislocation and revision after THA for ONFH, including younger age, higher body mass index, posterior surgical approach, and smaller femoral head size [18]. Differences in the temporal pattern of dislocation according to head diameter and improvements in implant materials and surgical practice have contributed to a gradual reduction in dislocation risk over time [19].

Collectively, Japanese ONFH research is characterized by the integration of nationwide epidemiologic infrastructure, MRI-based three-dimensional prognostication, biological investigation, and longitudinal surgical outcome analysis. By linking lesion-specific natural history to evidence-based treatment selection, this comprehensive approach has substantially influenced global strategies for both joint preservation and timely transition to arthroplasty in ONFH.

## DDH and reconstructive osteotomy

DDH is one of the principal causes of hip osteoarthritis in Japan accounting for approximately 74% (nearly three-quarters) of conditions leading to THA nationwide [20]. Because secondary osteoarthritis often progresses during young and middle adulthood, joint-preserving reconstructive osteotomy has long been a central strategy in Japanese hip surgery.

The evolution of periacetabular and proximal femoral osteotomies in Japan began soon after Chiari reported medial displacement osteotomy of the pelvis in 1955 [21]. In 1956, Nishio introduced transposition osteotomy of the acetabulum (TOA), mobilizing the acetabulum through a greater trochanteric approach [22]. Kawamura subsequently developed dome pelvic osteotomy, a modified Chiari-type curved osteotomy [23, 24]. In 1960, Mizuno devised tectoplasty, opening the outer iliac cortex laterally to augment coverage with bone graft, particularly for dislocated or high subluxated hips [25].

A major conceptual advance came with rotational acetabular osteotomy (RAO), initiated by Tagawa in 1968 [26]. Performed in the lateral position through a single incision using anterior and posterior intermuscular approaches, RAO allowed reorientation of the native acetabulum while preserving the posterior column. Comparative long-term analyses demonstrated that pelvic osteotomies were superior to proximal femoral varus or valgus osteotomies in preventing osteoarthritis progression in dysplastic hips. Surgical indications were progressively refined: RAO was preferred for spherical femoral heads with good congruency in abduction, particularly in hips with pre-osteoarthritis or early-stage osteoarthritis. Long-term follow-up studies demonstrated durable joint preservation exceeding 30 years when RAO was performed in appropriately selected early-stage cases [27]. Dome pelvic osteotomy was selected for flattened femoral heads and also demonstrated satisfactory long-term results [28–30], whereas tectoplasty remained indicated for dislocated or highly subluxated hips [25].

Further refinements of periacetabular osteotomies followed. In 2002, Hasegawa and colleagues described eccentric rotational acetabular osteotomy (eRAO), which medializes and distalizes the femoral head by shifting the acetabular rotation cengtre medially and superiorly, thereby reducing joint reaction force [31]. In 2005, Naito and colleagues introduced curved periacetabular osteotomy (CPO), modifying the Bernese periacetabular osteotomy (PAO) by using a curved osteotomy line and an intrapelvic approach to achieve less invasive redirection [32].

These procedures require exceptional technical precision. Surgeons must avoid intra-articular penetration, posterior column fracture, and neurovascular injury while achieving accurate reorientation [33]. Under-correction risks persistent instability, whereas overcorrection may induce femoroacetabular impingement (FAI). Determining the optimal magnitude and direction of acetabular reorientation therefore became a central challenge.

Japanese investigators addressed this issue early through three-dimensional (3D) imaging analysis. Beginning in the 1990 s, CT-based 3D evaluation enabled quantitative assessment of acetabular coverage, preoperative simulation of osteotomy lines, and estimation of postoperative joint contact pressures [34]. Patient-specific planning substantially improved reproducibility and safety, allowing individualized correction based on anatomical morphology rather than reliance on two-dimensional radiographs alone.

The introduction of computer-assisted orthopaedic surgery (CAOS) further enhanced accuracy. In 1997, Langlotz reported the first navigated PAO [35]. In 1999, Sugano and colleagues developed a CT-based navigation system in Japan incorporating surface registration of the pelvis and femur, enabling its application to pelvic and femoral osteotomies [36]. Unlike systems that merely display instrument position, this platform estimated acetabular fragment movement and updated the 3D bone model intraoperatively. Long-term follow-up of CT-based navigated RAO demonstrated high accuracy without severe complications such as infection, nonunion, osteonecrosis of the femoral head, or neurovascular injury [37].

During the 2010 s, commercial navigation systems were applied clinically. Akiyama reported safe and precise CPO using the Stealth Station system [38]. Takao and colleagues applied OrthoMap 3D navigation to RAO, enabling real-time visualization of osteotomy lines, chisel tip position, and target fragment orientation [39]. Notably, outcomes achieved by less-experienced surgeons approached those of experienced surgeons when navigation was used, suggesting that navigation may shorten the learning curve for technically demanding osteotomies [39].

Recent research has shifted from simply increasing acetabular coverage to determining how much coverage is optimal. 3D CT analyses revealed substantial interindividual variation in normal anterior and lateral coverage [40, 41]. Simulation studies demonstrated that even coverage approximating “normal” values may restrict hip range of motion (ROM) after RAO [42, 43]. Excessive anterior coverage has been associated with flexion impairment and FAI, adversely affecting long-term outcomes [44]. These findings underscore the need for patient-specific three-dimensional planning that integrates morphology, ROM, and predicted contact mechanics rather than targeting uniform radiographic angles.

For ONFH, proximal femoral osteotomies remain effective in selected young patients. Transtrochanteric rotational osteotomy promotes repair by repositioning the intact portion of the femoral head into the weight-bearing zone, and comparative studies have shown its durability relative to THA in patients under 50 years of age. CT-based navigation has also been applied to proximal femoral osteotomies, improving accuracy of correction and enabling achievement of target intact ratios with favorable mid- to long-term outcomes [45, 46].

In summary, the history of reconstructive osteotomy in Japan reflects a continuous integration of surgical innovation, biomechanical insight, and digital technology. From Chiari-derived techniques to RAO, eRAO, and CPO, and from two-dimensional planning to CT-based navigation and simulation, Japanese surgeons have progressively refined acetabular reorientation toward the pursuit of optimal, individualized coverage. This evolution represents a paradigm shift from structural augmentation alone to precision-guided joint preservation tailored to each patient’s three-dimensional anatomy and functional demands.

## Bearing surface innovations and implant longevity

Japan has played a pivotal role in advancing bearing surface technology and improving implant longevity in THA. A major milestone was the world’s first clinical application of highly cross-linked polyethylene (HXLPE), pioneered by Shikita [47]. Shikita also developed a monocrystalline alumina femoral head, which was presented at the 1978 SICOT Congress in Kyoto [48]. Retrieval analysis of ultra–high molecular weight polyethylene cross-linked with high-dose gamma irradiation after 24 years in vivo demonstrated substantially improved wear resistance, establishing the foundation for modern low-wear polyethylene bearings [49]. Earlier, long-term clinical results of polyethylene sockets combined with alumina ceramic heads had already shown favourable ten-year survivorship, supporting the transition toward harder, more wear-resistant femoral head materials [50]. These developments ultimately led to the now widely accepted standard articulation: ceramic-on–highly cross-linked polyethylene (CoHXLPE).

In parallel, fundamental materials research identified critical limitations of earlier ceramics. In vivo phase transformation and degradation of zirconia ceramic femoral heads after implantation were first reported in Japan [51]. This finding had major implications for material selection and stimulated improvements in ceramic processing and quality control worldwide.

Clinical investigations further clarified the long-term behaviour of modern bearings. Cementless third-generation alumina ceramic-on-ceramic (CoC) THA demonstrated excellent 11- to 14-year survivorship with minimal osteolysis, confirming that ceramic articulation can effectively suppress wear-induced bone resorption [52]. For HXLPE, comparative studies showed no significant difference in wear rates when articulated against cobalt-chromium or zirconia heads [53], and even enlargement of femoral head diameter from 26 to 32 mm did not increase linear wear at mid- to long-term follow-up [54]. These findings supported the use of larger heads to enhance stability without compromising durability, further consolidating CoHXLPE as a reliable contemporary standard.

Beyond bearing surfaces, Japanese investigators have addressed implant longevity through anatomical optimization for DDH, a condition characterized by distinctive femoral morphology. Three-dimensional CT analyses clarified the complex deformities of the dysplastic femur [55], leading to the development of custom-made femoral components [56] and modular neck systems enabling individualized anteversion adjustment [57]. Detailed 3D studies of proximal femoral geometry also contributed to the concept of anatomically shaped short stems for proximal fixation [58, 59]. For severe dislocation (Crowe type IV), cementless THA combined with subtrochanteric shortening osteotomy demonstrated favourable outcomes, allowing stable fixation despite extreme deformity [60].

Acetabular reconstruction in DDH has similarly evolved. Long-term follow-up of polyethylene sockets and ceramic heads emphasized the importance of adequate cup coverage and load transfer [50]. To quantify this, the concept of the cup center-edge (CE) angle was introduced to evaluate load-bearing coverage and determine whether structural bone grafting would be required [50]. Subsequent work demonstrated that press-fit–only cementless fixation could achieve stable outcomes without bulk structural grafting when adequate coverage was ensured [61]. More recently, porous titanium augments have been used effectively in primary THA for dysplasia or rapidly destructive coxopathy, expanding reconstructive options in cases of acetabular deficiency [62].

Hip resurfacing arthroplasty has also contributed important insights into bone preservation and implant durability. Dual-energy X-ray absorptiometry (DXA) studies showed preservation of proximal femoral bone mineral density after resurfacing [63], and vascular investigations clarified mechanisms protecting femoral head perfusion, thereby reducing ischaemic risk [64]. Clinical analyses demonstrated stable fixation even in cups without screw holes [65] and confirmed that the extent of osteonecrosis did not necessarily compromise survivorship compared with osteoarthritis in appropriately selected cases [66]. Long-term results in Asian populations have shown favorable durability [67], and studies evaluating postoperative sports participation demonstrated encouraging outcomes for activities such as jogging [68].

Collectively, these innovations—from HXLPE and advanced ceramics to anatomical implant design and biologically sound reconstruction—have significantly extended implant longevity. The integration of materials science, biomechanics, and 3D anatomical analysis has transformed THA from a pain-relieving procedure into a durable solution capable of meeting the functional demands of younger and more active patients.

## CT-based computer-assisted THA and osteotomy

Surgical robotics and navigation represent core technologies from the early era of CAOS. Advances in image processing and optical tracking enabled real-time intraoperative visualization of bone and instrument position, providing objective and quantitative guidance during surgery. In THA, inaccuracies in implant sizing or placement are directly associated with complications such as fracture, loosening, dislocation, impingement, wear, and leg length discrepancy. These risks created a strong demand to move beyond experience-dependent techniques toward reproducible, data-driven surgical support systems.

In the early 1990 s, ROBODOC was developed as one of the first surgical robots for orthopaedics, automatically milling the femoral canal according to a preoperative CT-based plan for cementless stems [69]. The system required rigid fixation of the femur and marker-based registration. In the late 1990 s, the development of optical tracking systems enabled frameless navigation. DiGioia and colleagues introduced CT-based navigation to measure and guide acetabular cup alignment intraoperatively [70]. However, their strategy placed the cup uniformly relative to the anterior pelvic plane (APP), without accounting for individual variations in pelvic tilt.

Sugano and colleagues were the first to clinically apply CT-based navigation [71] that guided not only the cup but also the femoral stem [72]. Their system demonstrated improved accuracy of cup alignment, reduction of outliers, and normalization of leg length and offset compared with conventional techniques [73]. Importantly, this approach extended beyond static alignment: intraoperative measurement of ROM using navigation, combined with analyses of deep flexion activities using open MRI and dynamic imaging, clarified the hip ROM required for daily activities—approximately 120° flexion, 40° extension, 40° external rotation, and 40° internal rotation at 90° flexion [72, 74–76]. Based on these data, the target was redefined as achieving cup and stem alignment that avoids impingement within this required functional ROM [77].

Crucially, rather than referencing the APP, Sugano’s group introduced cup placement relative to the functional pelvic plane (FPP), defined in the supine position as a functional “zero” pelvic orientation [78]. They were also the first to evaluate pelvic sagittal inclination in supine, standing, and sitting positions using three-dimensional CT [79] and to clarify long-term chronological changes over 10 to 20 years after THA [80, 81]. Postoperative dynamic motion analysis confirmed the validity of adjusting cup anteversion according to stem anteversion and implant design when positioning the cup relative to the FPP [82]. Even in patients with spinopelvic imbalance, including those exhibiting posterior pelvic tilt from supine to standing, this functional alignment strategy prevented impingement and dislocation as demonstrated by motion analysis studies [83]. In contrast, methods that adjust cup angle solely based on two-dimensional measurements of sacral slope or lumbar lordosis in standing and sitting positions do not account for femoral anteversion [84], despite evidence that stem anteversion significantly influences impingement risk [77, 85].

With the growing adoption of robotic-assisted THA, the concept has evolved from uniform target cup angles to individualized functional positioning. Enhanced navigation modes enable accurate stem placement, quantitative assessment of leg length, offset, and soft-tissue tension, and integration of femoral anteversion into cup positioning strategy [86]. CT-based robotic systems have demonstrated even greater angular precision for cup placement than conventional navigation [87], and robotic guidance has improved the accuracy of femoral component anteversion [86].

Numerous studies from Japan have validated the clinical utility of CT-based navigation. Compared with conventional techniques, CT-based navigation significantly improves cup alignment accuracy and reduces alignment outliers [72]. Accuracy is maintained even in minimally invasive THA approaches [88]. Navigation of the femoral side allows intraoperative evaluation of leg length discrepancy, offset, and ROM [73, 89], and the technology has proven applicable in revision THA [90]. In long-term follow-up of CoC THA, appropriate cup and stem positioning using CT-based navigation was associated with reduced dislocation and impingement-related complications, potentially contributing to improved implant survival beyond ten years [91]. Navigation has also demonstrated improved accuracy in hip resurfacing arthroplasty [92].

The evolution of THA navigation in Japan reflects a paradigm shift: from placing the cup at a fixed angle relative to the APP to individualized alignment relative to the FPP, incorporating stem anteversion and implant design to prevent impingement within the required functional ROM. Extensive investigations into femoral rotational alignment have further clarified reference axes and functional anteversion concepts [93, 94], and studies have shown that normalization of excessive anteversion in developmental dysplasia of the hip does not adversely affect postoperative rotation [95].

Beyond THA, CT-based navigation has been widely applied to osteotomies and pelvic fracture fixation, and it holds promise as an educational tool. Recent advances include automated CT-based preoperative planning using statistical atlases [96] and the extraction of additional imaging biomarkers, such as quantitative assessment of gluteal muscle volume and quality or whole-body muscle mass from preoperative CT data [97, 98]. As CAOS progresses, integration of artificial intelligence (AI) is expected to reduce planning burden, utilize latent imaging information, and further transition surgical support from merely achieving accuracy toward optimizing postoperative function.

## Future perspectives and conclusions

From a global perspective, Japan’s contribution to hip surgery extends far beyond conventional THA. It encompasses biomaterials innovation, CT-based navigation, spinopelvic biomechanics, complex reconstructive strategies, and importantly, computer-assisted osteotomy. The unifying philosophy has been precision restoration of anatomy and function rather than simple implant placement.

In bearing surface technology, Japanese investigators pioneered the clinical introduction of HXLPE and helped establish Co HXLPE as the modern standard articulation. Fundamental research clarifying in vivo degradation of zirconia ceramics influenced global material development. Long-term studies of CoC bearings demonstrated sustained suppression of osteolysis. These contributions collectively redefined durability in hip arthroplasty.

Equally transformative was Japan’s leadership in CT-based CAOS. The first clinical application of CT-based navigation guiding both acetabular and femoral components shifted THA from empiric alignment toward quantitative, patient-specific reconstruction. The introduction of the **FPP** concept, combined with stem-anteversion–adjusted cup alignment, anticipated today’s global focus on spinopelvic balance and functional positioning. Long-term analyses of pelvic sagittal tilt in supine, standing, and sitting positions further positioned Japan at the forefront of the hip–spine field.

However, Japan’s strength lies not only in arthroplasty but also in **joint-preserving osteotomy**. Rotational acetabular osteotomy, femoral osteotomy, and combined procedures for DDH have long been refined in Japan. The application of CT-based navigation to osteotomy allowed 3D correction planning, precise angular adjustment, and intraoperative verification of fragment orientation. This translated deformity correction from a two-dimensional radiographic estimation into a reproducible three-dimensional reconstruction.

Importantly, experience gained from osteotomy informed arthroplasty philosophy. Understanding acetabular coverage, femoral torsion, and load transfer in joint-preserving surgery directly influenced cup positioning strategies, CE-angle–based reconstruction concepts, and individualized femoral anteversion control in THA. Thus, osteotomy and arthroplasty development in Japan evolved synergistically rather than independently.

In complex DDH and high dislocation, Japanese surgeons integrated subtrochanteric shortening osteotomy with cementless THA, demonstrating that even severe deformities could be reconstructed with biomechanical fidelity. CT-based planning enhanced safety in these technically demanding cases. Beyond elective reconstruction, navigation has also been applied to pelvic trauma osteosynthesis, reinforcing its role across reconstructive hip surgery.

Looking ahead, the convergence of robotic assistance, AI, and advanced imaging analytics will likely expand Japan’s precision-based approach. Automated CT planning using statistical atlases, quantitative evaluation of muscle volume and quality, and integration of spinopelvic motion data suggest a future in which both arthroplasty and osteotomy are guided by comprehensive functional modeling. AI may assist in determining whether a patient benefits more from joint preservation (osteotomy) or replacement (THA), based on morphology, cartilage status, muscle condition, and predicted lifetime demand.

In conclusion, Japan’s contribution to hip surgery is distinguished by a holistic continuum:

- **Joint preservation through anatomically precise osteotomy**,
- **Durable reconstruction through advanced bearing surfaces**, and
- **Function-oriented arthroplasty through CT-based navigation and robotics**.

Rather than viewing osteotomy and arthroplasty as separate domains, Japanese research has integrated them within a single biomechanical framework centered on restoring native hip function. This comprehensive, anatomy-driven, and increasingly data-driven philosophy continues to shape the global evolution of hip reconstruction and will likely define its next era.

## Data Availability

No datasets were generated or analysed during the current study.
